# *rebuff* regulates apical luminal matrix to control tube size in *Drosophila* trachea

**DOI:** 10.1242/bio.036848

**Published:** 2018-09-15

**Authors:** Rachana R. Chandran, Aaron Scholl, Yuyang Yang, Lan Jiang

**Affiliations:** Department of Biological Sciences, Oakland University, 2200 N. Squirrel Road, Rochester, MI 48309, USA

**Keywords:** *rebuff*, Luminal matrix, Endocytosis, ObstA

## Abstract

The *Drosophila* embryonic tracheal network is an excellent model to study tube size. The chitin-based apical luminal matrix and cell polarity are well known to regulate tube size in *Drosophila* trachea. Defects in luminal matrix and cell polarity lead to tube overexpansion. Here, we address the novel function of the *rebuff* (*reb*) gene, which encodes an evolutionarily conserved Smad-like protein. In *reb* mutants, tracheal tubes are moderately over-elongated. Despite the establishment of normal cell polarity, we observed significantly reduced apical luminal matrix in *reb* mutants. Among various luminal components, luminal Obstructor-A (ObstA) is drastically reduced. Interestingly, ObstA is localized in vesicle-like structures that are apically concentrated in *reb* mutants. To investigate the possibility that *reb* is involved in the endocytosis of ObstA, we analyzed the co-localization of ObstA and endocytic markers in *reb* mutants. We observed that ObstA is localized in late endosomes and recycling endosomes. This suggests that in *reb* mutant trachea, endocytosed ObstA is degraded or recycled back to the apical region. However, ObstA vesicles are retained in the apical region and are failed to be secreted to the lumen. Taken together, these results suggest one function of *reb* is regulating the endocytosis of luminal matrix components.

## Abstract (precis)

**Summary:** Novel function of Smad-like protein Rebuff in regulating tube size of *Drosophila* trachea through endocytosis of luminal matrix components.

## INTRODUCTION

Distinct tube size is critical for the function of human tubular organs such as the lung, vascular system and kidney. Aberrant tube sizes lead to devastating human illnesses such as ischemic tissue injury and polycystic kidney disease. Studies in *Drosophila* trachea have shed light on mechanisms of tube-size regulation. The *Drosophila* trachea is the major airway consisting of branches with well-defined and consistent dimensions. Tracheal branches are formed by single layers of epithelial cells with the apical surface facing the luminal matrix and basal surface facing the surrounding tissues. The specification of tracheal tubes depends on signaling pathways, such as epithelial growth factor (EGF) and transforming growth factor (TGF-β) signaling, that are activated in a given group of cells (Llimargas and Casanova, 1999; Chen et al., 1998; Chihara and Hayashi, 2000). Branch specific signaling and fibroblast growth factor (FGF) signaling guide the migration of tracheal cells in stereotypical directions to form distinct branches. Once branch identities are specified, control of tube size is mediated by changes at the apical side of the tracheal cells (Beitel and Krasnow, 2000). The chitin-based apical luminal matrix and cell polarity are well-studied in tube-size regulation (Zuo et al., 2013). The apical luminal matrix contains the polysaccharide chitin, the chitin modifying enzymes Vermiform (Verm) and Serpentine (Serp) (Luschnig et al., 2006) and the chitin binding proteins Gasp (Tiklová et al., 2013), Knickkopf (Knk) (Moussian et al., 2006) and Obstructor-A (ObstA) (Petkau et al., 2012). Defective chitin synthesis leads to over-expanded tubes in both diameter and length (Devine et al., 2005; Araújo et al., 2005); loss of chitin-modifying enzymes Verm and Serp leads to over-elongated tubes (Luschnig et al., 2006); premature degradation of luminal matrix components by mutation in *obstA* gene results in over-elongated and slightly irregular tubes (Petkau et al., 2012).

The assembly of the luminal matrix depends on the apical secretion of cargos containing luminal components. COPI (Jayaram et al., 2008) and COPII (Förster et al., 2010) are involved in the early steps of apical secretion. Next, specific secretion pathways mediate the continuous secretion of distinct post-Golgi vesicles. For example, actin-MyoV and septate junctions (SJs) regulate the secretion of Gasp and Verm/Serp, respectively (Jayaram et al., 2008; Norum et al., 2010; Massarwa et al., 2009; Wu and Beitel, 2004). The luminal matrix is then maintained in the lumen before its clearance at the end of embryogenesis. The method through which the luminal matrix is maintained in the lumen remains largely unknown. Only recently, a report showed that Rab9 mediates the specific recycling of Serp, which in turn regulates tube size (Dong et al., 2013).

Cell polarity, including apical-basal and planar cell polarity (PCP), regulates tube-size. Apical-basal polarity regulates tube-size through polarized secretion as well as the expansion of apical membrane. Loss of function mutations in genes coding for most of basolateral septate junction components (Sinuous, Megatrachea, Kune-kune, Lethal giant larvae, Discs large, Scribble, the Na+/K+-ATPase, Neurexin IV, Lachesin and Varicose) lead to defective secretion of chitin modification enzymes (Verm and Serp), which subsequently causes over-elongated tubes (Wu and Beitel, 2004; Moyer and Jacobs, 2008; Paul et al., 2003; Llimargas et al., 2004; Behr et al., 2003). However, defects in two other SJ polarity proteins, Yurt and Coracle (Cora), cause tube over-elongation due to expanded apical membrane whereas the secretion of chitin modifying enzymes is normal (Laprise et al., 2010). Similarly, disruption of planar cell polarity (PCP) genes causes mild tube over-elongation resulting from an increased apical cell surface (Chung et al., 2009).

Here, we report a novel function of Rebuff (Reb), a Smad-like protein. Reb forms a subfamily with *Drosophila* Expansion (Exp), an uncharacterized *C. elegans* protein, C34E11.2c and the uncharacterized human protein Mothers against Dpp (Mad) homolog 3 (isoform X1). Both Reb and Exp are expressed in the multi-cellular branch dorsal trunk (DT) and are required for the secretion of chitin to the lumen, which is revealed by *exp, reb* double mutants (Moussian et al., 2015). In the *reb* single mutants we generated, we observed over-elongated tubes and reduced luminal components. Among them, ObstA showed the most obvious reduction. Instead of being secreted to the lumen, ObstA is apically concentrated in vesicle-like structures. We further analyzed the cellular localization of ObstA and we observed that ObstA is localized in endocytic vesicles including late endosome Rab7 as well as recycling endosome Rab11 and Vps26. These results suggest that in *reb* mutant trachea, endocytosed ObstA is degraded or is recycled back to the apical region. However, ObstA vesicles are retained in the apical region and are failed to be secreted to the lumen. Furthermore, overexpressing HA-tagged Reb protein (HA-Reb) in *reb* mutants at room temperature completely rescues *reb* mutant phenotype. Taken together, these results suggest that Reb is involved in regulating the endocytosis of luminal matrix components.

## RESULTS

### Identification of *Drosophila rebuff* (*reb*) gene and generation of *reb* null mutants

**Fig. 1.**
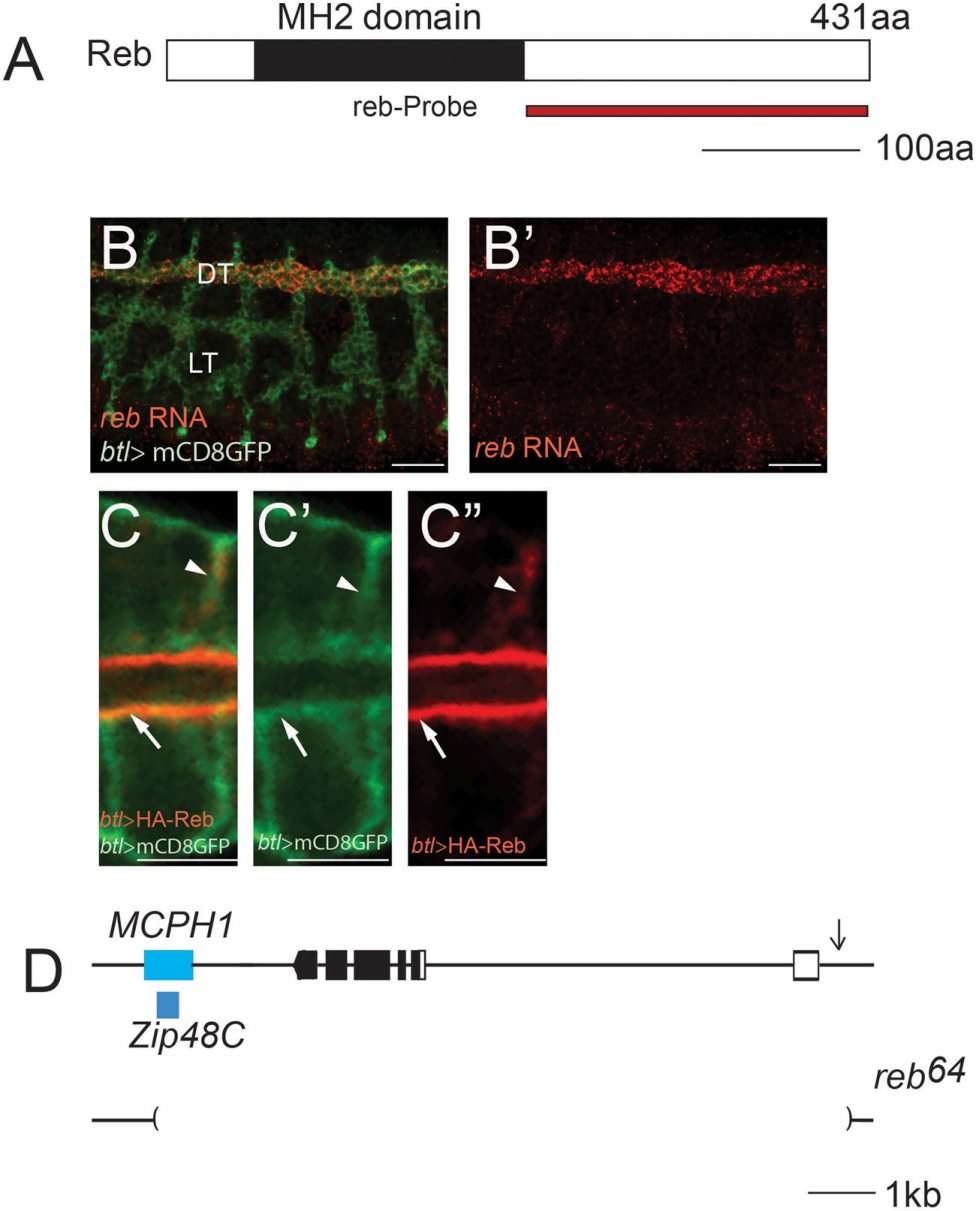
**Identification of the *Drosophila reb* gene and generation of *reb* null mutant *reb^64^*.** (A) Reb is a Smad-like protein containing a MH2 domain. A region (229-431aa) was used to generate *reb* probe. Black line represents 100 amino acids (aa). (B,B′) *reb* RNA (red) was localized in DT tracheal cells in wild-type *w^1118^* embryos and tracheal cells were labeled with mCD8GFP (green). DT, dorsal trunk (multicellular tube); LT, lateral trunk (unicellular tube). Scale bars: 50 μm. (C-C″) HA-tagged Reb protein (red in C and C″) and apical membrane (green in C and C′) was co-localized at the apical membrane (arrow) and the partial basolateral membrane (arrowhead) of the trachea cells. Stage 16 DT cells are shown. Cell membrane is outlined by mCD8GFP (C′). Scale bars: 5 μm. (D) Genomic structure of the *reb* gene locus. Boxes indicate exons: black boxes indicate coding sequence, connecting lines indicate introns. Blue boxes indicate the genomic locus of the two neighboring genes *MCPH1* and *Zip48C*. Brackets in lower lines indicate the region deleted in *reb^64^.* Black line represents 1 kb.

Our recent work showed that the *expansion* (*exp*) gene, which encodes a Smad protein, is required for tube-size regulation in *Drosophila* trachea in unicellular and intracellular branches (Iordanou et al., 2014). We identified Should be italicized as *CG13183* in an *in situ* hybridization screen for genes expressed in the trachea. It was named *rebuff* (*reb*) in a recent independent publication (Moussian et al., 2015). The *reb* gene encodes an N-terminal Mad-homology 2 (MH2) domain protein (Fig. 1A). Phylogenetic analysis of the MH2 domain of Reb shows that it forms a subfamily with *Drosophila* Expansion, an uncharacterized *C. elegans* protein, C34E11.2c, and the uncharacterized human protein Mothers against Dpp homolog 3 (isoform X1). To define the role of *reb* gene in tube-size regulation, we generated a *reb* null mutant by imprecise excision of a P-element *MCPH1^NP6229^*, located at 5′ upstream of the *reb* gene. We isolated *reb^64^*, which removes the entire *reb* locus as well as disrupting two neighboring genes, *MCPH1* and *Zip48C* (Fig. 1D). We use *reb^64^* as a *reb* null mutant in the following experiments. Similar to the BDGP reported expression pattern of this gene, RNA *in situ* hybridization shows that *reb* is expressed in the DT, a multicellular tube from embryonic stage 14 to 17 (Fig. 1B,B′). We generated a Reb antibody and unfortunately it did not successfully detect the Reb protein in embryos by immunohistochemistry. To visualize Reb protein localization, we expressed HA-Reb together with membrane marker mCD8-GFP specifically in trachea via *btl-gal4* (Ohshiro et al., 2002). Reb was highly concentrated at the apical membrane (arrowheads in Fig. 1C-C″) and weakly expressed at the basal lateral cell membrane (arrows in Fig. 1C-C″). It is consistent with the Reb expression pattern published recently (Moussian et al., 2015).

### Multicellular tubes are over-elongated in *reb* mutants

**Fig. 2.**
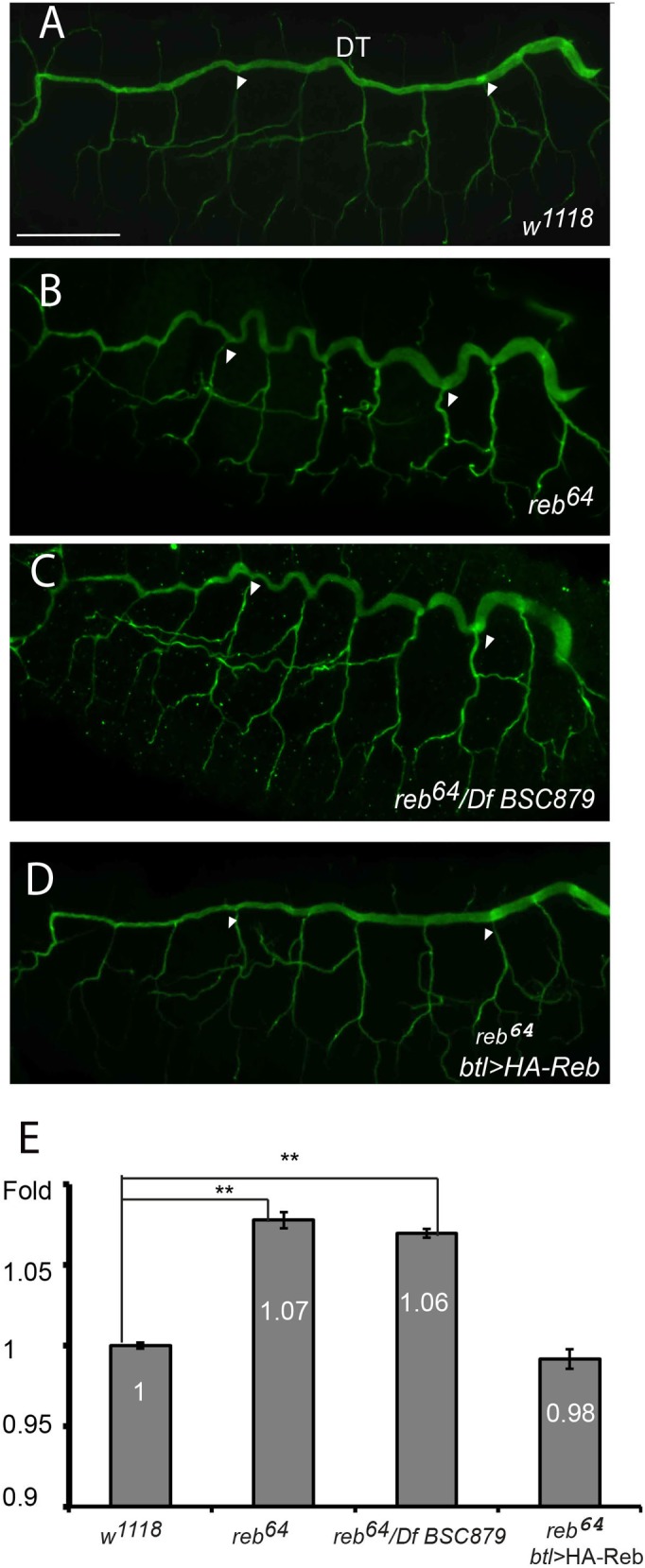
**The multicellular tube DT is over-elongated (curly phenotype) in *reb* mutants.** Stage 16 wild-type *w^1118^*, *reb^64^* mutants, transheterozygotes *reb^64^* over *Df BSC879,* and *reb^64^* mutants with HA-Reb expressed in trachea using *btl-gal4* at room temperature were immunostained with a chitin probe. (A) Wild-type DT was relatively straight. (B) *reb^64^* mutant DT was curly. (C) Transheterozygous *reb^64^*/*Df BSC879* was curly*.* (D) *reb^64^ +btl>*HA-Reb was relatively straight. (E) Measurement of the fold changes of various genotypes. Length of DT tracheal segments 4-8 was measured (between white arrowheads) and normalized against the overall length of the embryo. Error bars represent standard error. Ten embryos for each genotype were used. Fold changes between various genotypes were calculated. Scale bar: 50 μm. Significant difference was shown by Student's *t*-test (**P<0.01).

To reveal the role of *reb* gene in tube-size regulation, we analyzed the trachea with a probe detecting chitin, a fibrous substance consisting of polysaccharides secreted to the tracheal lumen, in both wild type and *reb^64^*. The multicellular tube DT was curly and slightly irregular in *reb^64^* mutants (Fig. 2B) compared to the relatively straight wild-type DT (Fig. 2A). To further confirm the phenotype, we also analyzed DT in transheterozygotes of *reb^64^* over *Df BSC879*, a deficiency line that has been used to create *reb, exp* double mutant as described (Moussian et al., 2015). Not surprisingly, we observed similar curly DT phenotype (Fig. 2C). Despite this phenotype, the tracheal network exhibited normal branching patterns, and no other obvious defects were observed. A curly phenotype usually suggests tube over-elongation (Beitel and Krasnow, 2000). Therefore, we quantified the length of DT (between white arrows in Fig. 2) in ten stage-16 embryos. Since *Drosophila* embryos have different embryonic length depending on genotypes, we normalized the DT length to the overall embryonic length. The fold changes between wild-type and *reb* mutant were calculated. We observed ∼7% and ∼6% increase in *reb^64^* and *reb^64^/Df BSC879* mutants compared to wild-type control (Fig. 2E).To confirm that the tube over-elongation phenotype in *reb* mutants was due to *reb*, as opposed to *MCPH1*, *Zip48C* or background mutations, we expressed HA-Reb throughout the trachea in *reb^64^* mutants at room temperature. Specifically, we crossed *reb^64^/CyO P[w^+^, twi–GFP]_;_UAS-HA-Reb* and *reb^64^/CyO P[w^+^, twi–GFP]; btl-gal4* lines to overexpress HA-Reb in trachea and analyze tracheal lumen in *reb* mutant background by choosing GFP negative embryos. As expected, we observed almost complete rescue of DT tube length (Fig. 2D,E). Thus, *reb* acts autonomously in the trachea to control tube size.

### *reb* is not required for the establishment of cell polarity

**Fig. 3.**
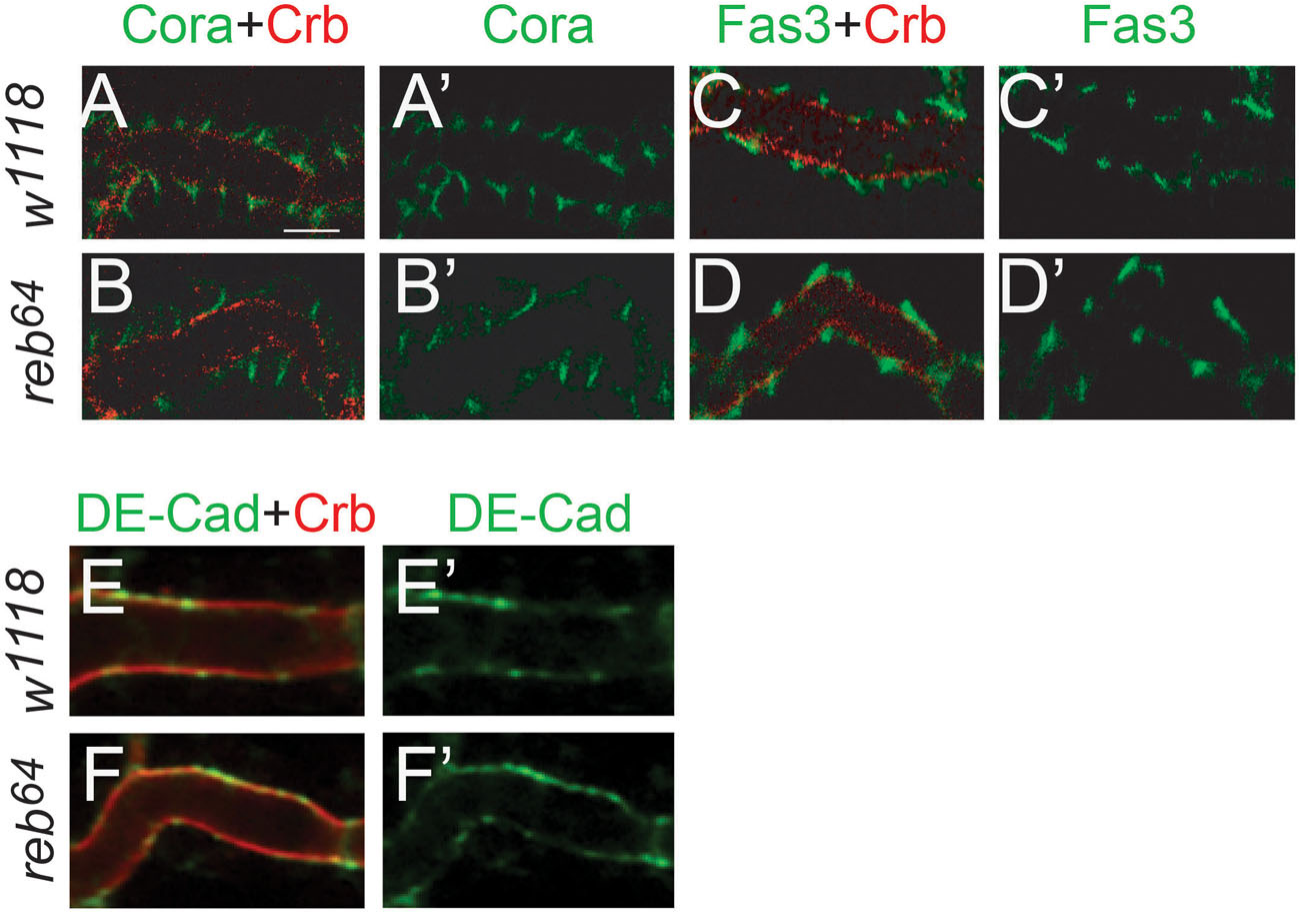
***reb* is not required for the establishment of cell polarity.** Apical membrane marker Crumbs (Crb in red), SJ marker Cora (in green), Fas3 (green) and adherens junction marker DE-cadherin (DE-Cad in green) were analyzed in stage 16 *reb* mutant and wild-type *w^1118^*. Apical Crb and SJ marker Cora and Fas3 were correctly localized in DT in *reb* mutant (B,B′,D,D′) similar to wild-type control (A,A′,C,C′). Adherens junction marker DE-cad was correctly localized in *reb* mutant DT (F,F′) as wild-type DT (E,E′). Scale bar: 5 μm. Ten embryos of wild-type and *reb* mutant were imaged for each staining.

The *Drosophila* trachea is composed of polarized epithelial cells. Cell polarity plays a significant role in tube-size regulation, particularly tube length. The basolateral septate junction, analogous to the vertebrate tight junction, forms a diffusion barrier to prevent the exchange of solutes across epithelia (Tepass et al., 2001). A compromised SJ leads to tube over-elongation either due to defective secretion of chitin-modification enzymes (Verm and Serp), such as loss of function mutation in genes coding for *Drosophila* Claudins, Na+/K+ ATPase, and Lachesin (Paul et al., 2003; Llimargas et al., 2004; Wu et al., 2004; Nelson et al., 2010); or due to increased apical membrane size such as loss of function mutation in genes coding for Yurt and Cora (Laprise et al., 2010). To test whether *reb* regulates cell polarity, we analyzed the trachea using markers for apical membrane Crumbs (Crb), adherens junction (AJ, DE-cadherin) and multiple SJ markers (Discs large, Cora, Fas3, Nrv2.1) by an immunohistochemistry approach. Apical membrane Crb (red in Fig. 3B,D,F compared to A,C,E), AJ component DE-cadherin (green in Fig. 3F′ compared to 3E′) and SJ components were correctly localized in *reb* mutant as in wild-type trachea, and SJ components Fas3 (green in Fig. 3D′ compared to C′) and Cora (Fig. 3B′ compared to A′) were shown. Therefore, *reb* is not required for establishing or maintaining cell polarity in trachea cells.

### Reb likely does not play a significant role in regulating TGF-β signaling

**Fig. 4.**
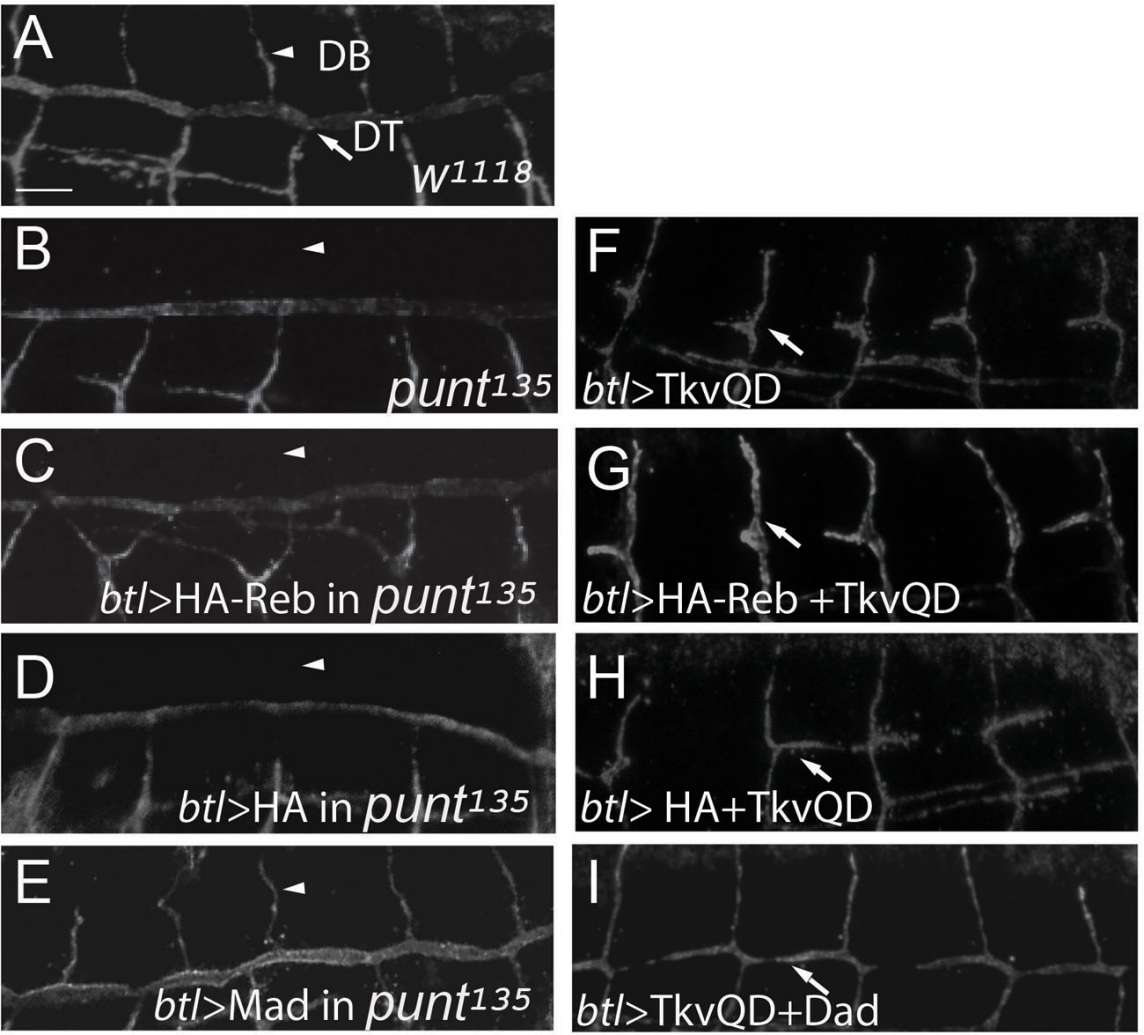
**Reb does not play a significant role in regulating TGF-β signaling.** Stage 15 embryos were stained with anti-Uif to label the apical membrane. (A) DT and DB form normally in wild-type trachea. (B) DB did not migrate out in zygotic *punt^135^* mutants. (C) Similar to *punt^135^* mutants, HA-Reb overexpression in trachea did not rescue the DB migration defects. (D) Similar to *punt^135^* mutants, HA overexpression in trachea did not rescue the DB migration defects. (E) Mad overexpression in *punt^135^* mutant trachea significantly rescued DB migration defect. (F) TkvQD overexpression in trachea led to DT formation defect. (G) Coexpression of HA-Reb and TkvQD in trachea had similar DT formation defect as TkvQD expression alone. (H) Coexpression of HA and TkvQD in trachea had similar DT formation defect as TkvQD expression alone. (I) Coexpression of Dad and TkvQD in trachea significantly rescued DT formation defect. Arrowheads, dorsal branch (DB); arrows, DT. Scale bar: 10 μm. Ten embryos of each genotype were imaged.

Reb belongs to the Smad family of proteins, which usually function as components or regulators [such as Mad and Daughters against decapentaplegic (Dad)] of TGF-β signaling (Kamiya et al., 2008). To test this possibility, we analyze the modifying effect of *reb* on TGF-β signaling mutant phenotype by expressing HA-Reb in constitutively active or loss of function TGF-β signaling mutants. In addition, a negative regulator of the pathway, Dad and a positive component, Mad, were expressed in constitutively active or loss of function TGF-β signaling mutants respectively as positive controls to show the effectiveness of the assay. In addition, HA was expressed in these signaling mutants as a negative control. If Reb regulates TGF-β signaling, expressing HA-Reb protein in these mutants will either suppress or enhance the mutant phenotype. Trachea expression of TkvQ253D, which over-activates TGF-β signaling, leads to DT migration defects (arrow in Fig. 4F) compared to the wild-type control (arrow in Fig. 4A). As expected, tracheal expression of a negative regulator, Dad, together with TkvQ253D significantly rescued the DT migration defect (arrow in Fig. 4I). However, co-expressing of HA-Reb together with TkvQ253D did not suppress the DT migration defect (arrow in Fig. 4G). Not surprisingly, as a negative control, co-expressing of HA and TkvQ253D had no effects (arrow in Fig. 4H).

TGF-β zygotic loss of function *punt^135^* mutants had defects in DB formation (arrowhead in Fig. 4B). As expected, Mad overexpressing in *punt^135^* mutants significantly rescued the DB migration defect (arrowhead in Fig. 4E). If Reb functions as a positive regulator of the pathway, we expect that the tracheal expression of HA-Reb likely rescues the DB migration defect of *punt^135^* mutants. However, expressing HA-Reb in *punt^135^* mutant trachea did not rescue DB migration defect (arrowhead in Fig. 4C). As expected, HA did not show any rescue either (arrowhead in Fig. 4D). Taken together, these genetic interaction assays indicate that Reb may not play a significant role in TGF-β signaling. However, the lack of positive genetic interaction cannot rule out the possible biochemical interaction of the Reb and TGF-β signaling components. Also, our genetic interaction assay may not be sensitive enough to reveal any relatively minor role of Reb in TGF-β signaling.

### Luminal matrix components are reduced in the lumen in *reb* mutants

**Fig. 5.**
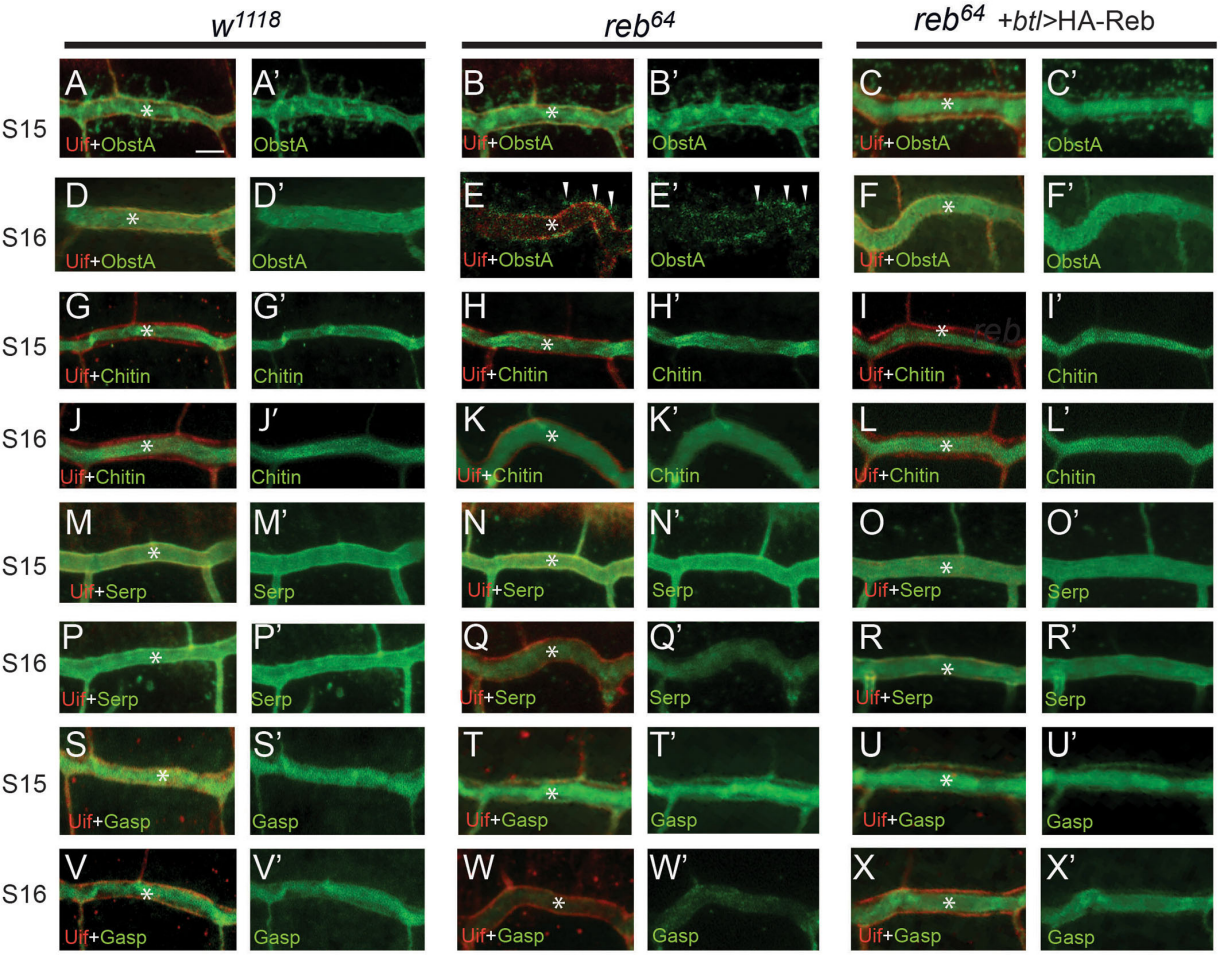
***reb* is required for the maintenance of apical matrix components.** Localization of luminal components ObstA, Chitin, Serp and Gasp were analyzed in wild-type, *reb^64^* mutant and *reb^64^* mutant trachea with tracheal expression of HA-Reb construct. Stage 15 and 16 DTs were shown. (A-C′) Apical membrane was labeled with Uif (red). At stage 15, luminal protein ObstA (green) was secreted normally to the DT lumen (*) in wild-type (A-A′), *reb* mutant trachea (B-B′) and *reb* mutant trachea with HA-Reb expression (C-C′). (D-F′) At stage16, ObstA was localized in lumen (*) in wild-type trachea (D-D′), significantly reduced in lumen in *reb* mutant (E-E′), but was localized in vesicles that were apically concentrated (arrowheads in E-E′). ObstA is normally localized in *reb* mutant trachea with HA-Reb overexpression (F-F′). (G-I′) At stage 15, chitin was secreted normally to the DT lumen (*) in wild-type (G-G’), *reb* mutant (H-H′) and *reb* mutant trachea with HA-Reb overexpressing (I-I′). (J-L′) At stage16, luminal chitin was localized mainly in lumen (*) in wild-type trachea (J-J′), slightly reduced in lumen in *reb* mutant (K-K′) and normally localized in *reb* mutant trachea with HA-Reb overexpression (L-L′). (M-R′) At stage 15, Serp was secreted normally to the DT lumen (*) in wild-type (M-M′), *reb* mutant (N-N′), *reb* mutant with tracheal HA-Reb overexpression (O-O′). AT stage16, luminal Serp was localized mainly in lumen (*) in wild-type trachea (P-P′), reduced in *reb* mutant (Q-Q′) and normally localized in *reb* mutant trachea with HA-Reb overexpression (R-R′). (S-U′) At stage 15, Gasp was secreted normally to the DT lumen (*) in wild-type (S-S′), *reb* mutant (T-T′), *reb* mutant with tracheal HA-Reb overexpression (U-U′). (V-X′) At stage16, luminal Serp was localized mainly in lumen (*) in wild-type trachea (V-V′), reduced in *reb* mutant (W-W′) and normally localized in *reb* mutant trachea with HA-Reb overexpression (X-X′). Scale bar: 5 µm. Ten embryos of respective genotypes were imaged for each staining.

The apical luminal matrix is required to prevent tube over-expansion (Swanson and Beitel, 2006). To test the effect of *reb* on the apical luminal matrix, we analyzed luminal components (chitin binding proteins, ObstA and Gasp, a chitin deacetylase, Serp and a polysaccharide, chitin) by immunohistochemistry and a probe recognizing polymerized chitin at stages 15 and 16. We observed similar localization of ObstA (compare Fig. 5B,B′ to Fig. 5A,A′), chitin (compare Fig. 5H,H′ to Fig. 5G,G′), Serp (compare Fig. 5N,N′ to Fig. 5M,M′) and Gasp (compare Fig. 5T,T′ to Fig. 5S,S′) in *reb* mutants and wild type respectively at stage 15. However, at stage 16, we observed that ObstA was drastically reduced in the lumen (* in Fig. 5E,E′) in *reb^64^* whereas it was properly localized in wild-type control (Fig. 5D,D′). Interestingly, ObstA was localized in vesicles which were apically concentrated (arrowheads in Fig. 5E,E′). In addition, luminal reduction of Serp (Fig. 5Q,Q′), and Gasp (Fig. 5W,W′) was also observed in *reb^64^* mutants compared to wild type (Fig. 5P,P′,V,V′). Nevertheless, the chitin level had only minor reduction (Fig. 5K,K′) compared to wild type (Fig. 5J,J′). We measured average chitin intensity in tracheal segment 7 in ten *reb* mutant and wild-type embryos respectively and we observed that chitin level in *reb* mutant is reduced ∼9.6%±0.5% compared to wild-type control. These observations suggest that Reb has a novel function in regulating tube size through the maintenance of luminal matrix components, in particular ObstA. To further demonstrate that the luminal matrix defect observed in *reb^64^* mutant is caused by the absence of *reb* gene, we overexpressed HA-Reb in *reb^64^* mutant trachea via *btl-gal4* at room temperature and we observed normal ObstA (Fig. 5C,C′,F,F′), chitin (Fig. 5I,I′,L,L′), Serp (Fig. 5O,O′,R,R′) and Gasp (Fig. 5U,U′,X,X′) in stage 15 and 16 trachea. The complete rescue of the luminal defect in *reb^64^* mutant trachea by HA-Reb at stage 16 further confirmed that *reb* is required for luminal matrix maintenance in trachea. Despite the progress made toward the secretion of luminal components, relatively little is known about the maintenance of the luminal matrix.

### ObstA are localized in endocytic vesicles in *reb* mutant trachea

**Fig. 6.**
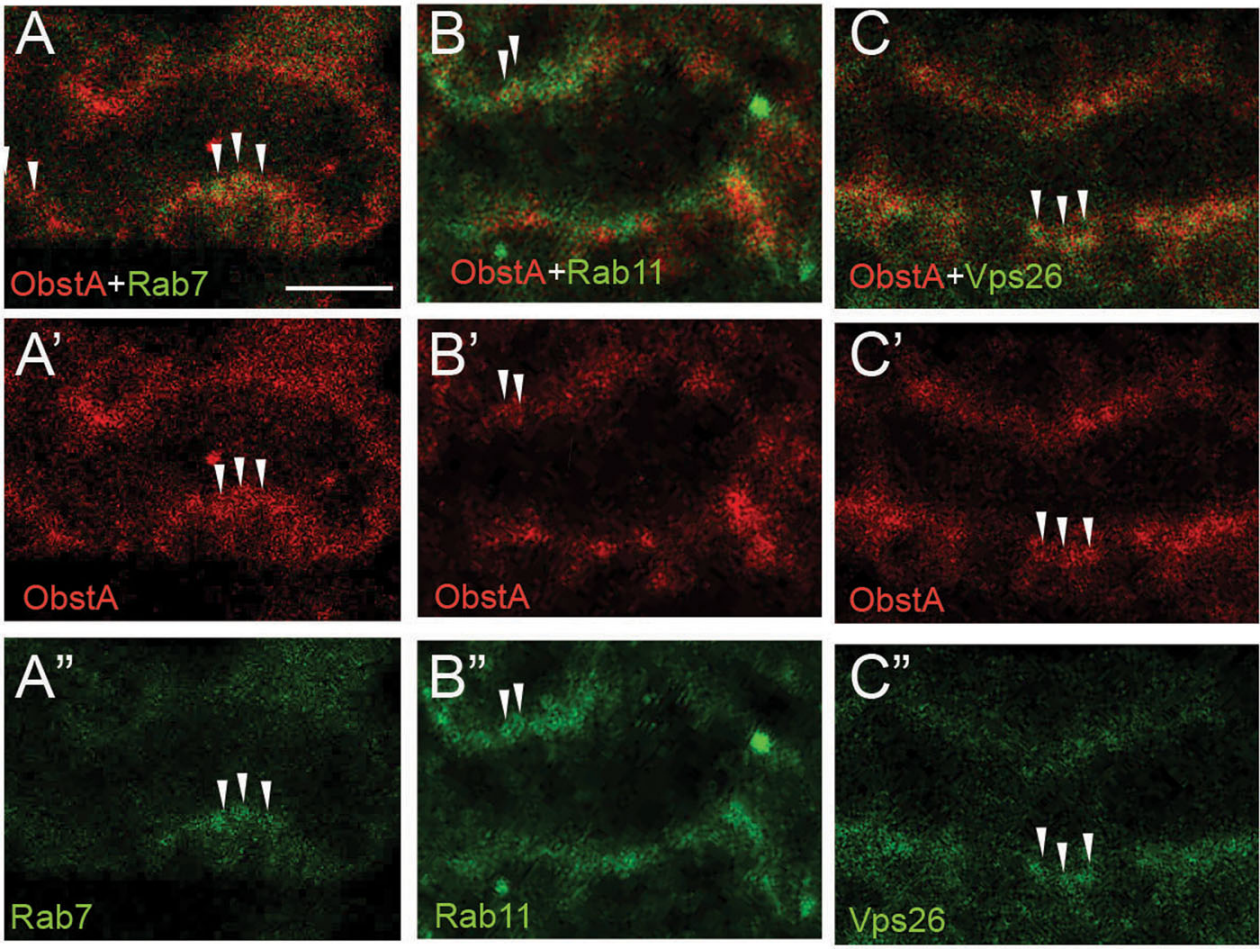
**ObstA is localized in endocytoic vesicles in *reb* mutant trachea.** Stage 16 *reb* mutant embryos were stained with antibodies of ObstA (red) and endocytic markers (green). (A-A″) Apically concentrated ObstA vesicles show overlap with late endosome Rab7 (arrowheads). (B-B″) Apically concentrated ObstA vesicles show overlap with recycling endosome Rab11 (arrowheads). (C-C″) Apically concentrated ObstA vesicles show overlap with recycling retromer Vps26 (arrowheads). Scale bar: 5 µm. Five embryos of *reb* mutant were imaged for each staining.

ObstA is a luminal protein that regulates the stability of the luminal matrix (Petkau et al., 2012). Therefore, the reduction of other luminal matrix components could be due to the lack of luminal ObstA in *reb* mutant trachea. Recently, a study showed that Rab9-mediated endocytic recycling is required for the maintenance of luminal Serp (Dong et al., 2013). It suggests that a diverse recycling pathway might be required to maintain distinct luminal matrix components. Interestingly, in *reb* mutant trachea, ObstA was localized in vesicles that were apically concentrated. To investigate the possibility that *reb* is involved in the endocytosis of ObstA, we analyzed the co-localization of ObstA vesicles and endocytic vesicles including late endosome Rab7, recycling endosome Rab11, and recycling retromer Vps26. Rab7 (Hyttinen et al., 2013; Jager et al., 2004) mediates the degradation of endocytosed molecular in lysosome, whereas the recycling endosome Rab11 (Ullrich et al., 1996) and retromer (Wang et al., 2014; Seaman, 2012) retrieve proteins from endosomes, thereby preventing the degradation of these proteins in lysosomes. Interestingly, we observed co-localization of ObstA and Rab7 (arrowheads in Fig. 6A-A″), ObstA and Rab11 (arrowheads in Fig. 6B-B″) and ObstA and Vps26 (arrowheads in Fig. 6C-C″). These results suggest that in *reb* mutant trachea, internalized ObstA could be degraded in lysosomes mediated by Rab7, or be recycled back to the apical region mediated by Rab11 and retromer. However, these recycled vesicles are retained in the apical region but not secreted to the lumen. Taken together, these results indicate that *reb* regulates luminal matrix to control tube size through the endocytosis pathway. However, the mechanisms through which *reb* regulates endocytosis remain to be further investigated.

## DISCUSSION

Here we revealed a novel function of a Smad-like protein Reb in regulating tube size possibly through the endocytosis pathway. Very recently, an independent study suggests that Exp/Reb regulates the deposition of chitin into the lumen. However, in our *reb* single mutant, only minor reduction of chitin deposition was observed. Therefore, the analysis of this *reb* single mutant allows us to investigate additional functions of Reb distinct from its role in the secretion of chitin. Interestingly, we observed drastic reduction of another luminal component ObstA in *reb* mutants. ObstA regulates the stability of other luminal matrix components, therefore, the reduced luminal matrix is likely due to the significant loss of luminal ObstA.

Interestingly, we observed apically concentrated ObstA vesicles in *reb* mutant trachea. These vesicles are co-localized with late endosomes and recycling endosomes. These results suggest that in *reb* mutants the internalized ObstA is either degraded or is recycled. However, the recycled ObstA retained apically and failed to be secreted to the lumen. Therefore, Reb is likely required for the recycling of luminal matrix components to the lumen and not to be degraded. We speculate that Reb could interact with membrane fusion machinery at the apical membrane to release the luminal matrix to the lumen.

Reb contains an N-terminal domain similar to the MH2 domain characteristic of Smad proteins, which mediates the effects of the TGF-β signaling pathway. However, our genetic interaction assays suggest that Reb may not function as a typical Smad protein to regulate the TGF-β pathway. Instead, it potentially regulates the recycling of luminal matrix and the activation of Moe. Another member of the Reb subfamily, Exp, does not function as a typical Smad protein either. Recently, the crystal structure of the MH2 domain of the Exp domain was determined. Compared to the conserved MH2 domain of Smad, the Exp MH2 domain has the addition of a helical region and the remodeling of a protein-interaction site (Beich-Frandsen et al., 2015). Therefore, the Reb subfamily of proteins belongs to a novel subfamily of the Smad superfamily and function as atypical Smads to regulate cellular processes and signaling pathways other than TGF-β signaling. The results presented here provide novel insights into the cellular mechanisms of Reb protein in tube size regulation and expand our general knowledge about this novel subfamily. The identification of Reb-interacting proteins and elucidation of protein domains involved by biochemical approaches will further reveal molecular mechanisms of this protein.

## MATERIALS AND METHODS

### Fly strains

Experiments utilized *W^1118^* as the control strain. *reb^64^* was generated by P element imprecise excision. *UAS-HA-reb* transgenic line was generated in our lab utilizing standard germline transformation. Other flies including *UAS- TkvQ253D*, and *UAS-mCD8-GFP, punt^135^, Rab11^93B^, Rab11^J20^* were from Bloomington Stock Center (Bloomington, USA). To generate deletion mutants of *reb*, approximately 100 *P{GawB}MCPH1^NP6229^* virgin females were crossed to about 100 jump starter *yw; P[w^+^;* Δ*2-3]* males. The resultant male progenies (about 200) w; *P{GawB} MCPH1^NP6229^/P[w^+^;* Δ*2-3]* were then mated with *yw; Pin/Cyo* virgin females (about 200). Individual white-eyed males from the F2 generation (400) were selected and backcrossed to *y w; Pin/CyO P[w^+^; twi–GFP]* females to establish stocks. We sequenced genomic DNA that was PCR-amplified from mutant alleles to determine the deletion breakpoints. In doing so, *Reb^64^* mutants were isolated. Homozygous mutant individuals were identified in genetic experiments by the absence of *GFP* expression from *CyO P[w^+^; twi–GFP]*.

### *In vitro* transcription and generation of *reb* RNA probe

A *reb* RNA probe was generated through *in vitro* transcription. PCR amplification of *reb* cDNA cloned into the pFlc-1 vector was carried out using the SK-30 (5′ GGG-TAA-CGC-CAG-GGT-TTT-CC 3′) and SK-Met (5′ ATG-ACC-ATG-ATT-ACG-CCA-AGC 3′) primers. To generate an antisense digoxigenin-labeled RNA probe, 50 µl of PCR product was ethanol precipitated, resuspended in DEPC-treated H2O and transcribed using T3 (Promega, Madison, USA) polymerase. The reactions were performed at 37°C for 3 h and contained the following: 1× transcription buffer, 10 mM DTT, 1× nucleotide labeling mix (Roche, Basel, Switzerland) containing dig-UTP, 20 units RNasin (Promega), and 8 units of RNA polymerase in a 10 µl final volume. RNA probes were DNase-treated with 1 µl of RQ1 DNase (Promega) for 30 min at 37°C. Probes were ethanol precipitated and resuspended in 100 µl of probe resuspension buffer (50% formamide, 5 mM Tris–HCl pH 7.5, 0.5 mM EDTA and 0.01% Tween-20).

### Fluorescence *in situ* hybridization

Approximately 500 btl>mCD8-GFP embryos (encompassing stages 12–17) were hybridized with the *reb* RNA probe. Probe was diluted 1:100 in hybridization buffer (50% glycerol, 4× SSC, 5% dextran sulfate, 0.01% Tween-20) and hybridization was performed in a total volume of 100 µl at 55°C O/N. Hybridized embryos were blocked in 0.5% blocking buffer (Perkin Elmer, Waltham, USA) and incubated for 1 h in anti-dig-POD antibody (Roche) which was diluted 1:50 in 0.5% blocking buffer. After 3×10 min PT (1× PBS, 0.1% Tween-20) washes, embryos were incubated for 2 h in TSA-Cy3 diluted (1:50) in amplification diluent (Perkin Elmer). TSA reaction was stopped by following with 3×10 min PT washes. *In situ* hybridization was followed by immuno-staining. The 1° antibodies rat anti-Trh and chicken anti-GFP at 1:100 dilution and 2° antibodies anti-chicken-488 and anti-Rat-647 at 1:100 dilution were used for the immunolabeling of tracheal membrane and nucleus.

### Generation of *UAS-HA* and *UAS-HA-reb* transgenic strains

To generate HA-tagged *reb* fragments, *reb* cDNA fragment without the ATG start codon was PCR amplified from *reb* cDNA clone RE66796 and cloned into pCR8/GW/TOPO (Invitrogen). The inserts were then cloned into pTHW, a UAS-HA-tag-containing Gateway compatible vector (https://dgrc.bio.indiana.edu), using Gateway LR Clonase II plus (Invitrogen). The pTHW and UAS-HA-*reb* constructs were introduced into the *Drosophila* germline by microinjection respectively to generate *UAS-HA* and *UAS-HA*-*reb* transgenes.

### Immunohistochemistry

Regular staining: embryos were subjected to immunostaining immediately or within a few days after fixation with 4% formaldehyde for 20 min. For regular immunostaining, embryos were washed in PT (1× PBS, 0.1% Triton X-100) 3×10 min and blocked in PBT-NGS [1× PBS, 0.1% BSA, 0.1% Triton X-100, 5% Normal Goat Serum (NGS)] for 30 min. Embryos were incubated in 1° antibody diluted in PBT-NGS overnight at 4°C, washed 3×10 min, then 3×20 min in PBT and blocked for 30 min in PBT-NGS. Embryos were then incubated in 2° antibody diluted in PBT-NGS (1:100) for 2 h at room temperature and washed 3×10 min followed by 3×20 min in PBT. Embryos were mounted in 100% glycerol then imaged using a Nikon Eclipse Ti confocal microscope.

Tyramide signal amplification (TSA) immunostaining: fixed embryos were washed in PT (1× PBS, 0.1% Trition X-100) 3×10 min and blocked in PBT-NGS (1× PBS, 0.1% BSA, 0.1% Trition X-100, 5% NGS) for 30 min. Embryos were incubated in 1° antibody diluted in PBT-NGS overnight at 4°C, washed 3×10 min then 3×20 min in PBT, and blocked for 30 min in PBT-NGS. Embryos were then incubated in Biotin labeled 2° antibody (Vector Laboratories, Burlingame, USA) diluted in PBT-NGS (1:300) for 1 h at room temperature and washed 3×10 min then 3×20 min in PBT. Embryos were incubated in streptavidin-conjugated HRP (Perkin Elmer) diluted in 1× PBT (1:100), and washed 3×5 min then 3×10 min in PBT. Treated with TSA fluorescein (TSA cyanine 3/TSA cyanine 5/TSA fluorescein; 1:50 dilution in amplification diluent) for 10 min in dark, washed 3×10 min, then 3×20 min in PBT. Embryos were mounted in 100% glycerol and images were obtained using a Nikon Eclipse Ti confocal microscope.

### Antibodies used for immunostaining

The chitin binding probe was prepared in our laboratory. pYZ205, a 6× His-SNAP-CBD plasmid [provided by New England Biolabs (NEB) upon request], was transformed to an *E.coli* strain. 6× His-SNAP-CBD expression was induced using 1 mM IPTG and purified by affinity chromatography using Nickel resin. To label the purified chitin binding probe with SNAP tag, SNAP-surface Alexa Fluor 488 (NEB; cat# S9129S) was added and incubated for 1 h at 37°C. The fluorophore labeled probe was then dialyzed overnight in 1× PBS containing 1 mM DTT to remove unreacted substrates. The labeled probe was stored at −20°C in 50% glycerol. List of 1° antibodies used are given below.

The following 1° antibodies were used: Chicken anti-GFP (1:200) (Abcam), Guinea Pig anti-Uif (1:500) (Zhang and Ward, 2009), Mouse anti-Crb (1:2) [Developmental Studies Hybridoma Bank (DSHB), Iowa City, USA], Rat anti-Trh (1:100) (Ward et al., 1998), Mouse anti-Gasp (1:5) (DSHB), Rabbit anti-Obst-A (1:200) (Petkau et al., 2012), Chicken anti-GFP-IgY (1:100) (Invitrogen), Rat anti-DE-Cad (1:50) (DSHB), Guinea Pig anti-Cora (1:100) (Lamb et al., 1998), Rabbit anti-Pio (1:100) (Bokel et al., 2005), Rabbit anti-Verm (1:100) (Luschnig et al., 2006), Rabbit anti-Serp (1:100) (Luschnig et al., 2006), Rabbit anti-HA (1:100) (Invitrogen), Rabbit anti-Sinu (1:200) (Wu et al., 2004), Rat anti-Vari (1:200) (Wu et al., 2007), Rabbit anti-Kune kune (1:200) (Nelson et al., 2010), Guinea Pig anti-Yurt (1:50) (Laprise et al., 2009), Guinea Pig anti-Vps26 (1:100) (Wang et al., 2014), Mouse anti-Rab7 (1:30), Mouse anti-Rab11 (1:50) (BD Transduction Laboratories, San Jose, USA), Mouse anti-Fas3 (1:50) (DSHB), Mouse anti-Discs large (1:1) (DSHB), Rabbit anti-Nrv2.1 (1:200) (Wu et al., 2007), and Mouse anti-Spectrin (1:20) (DSHB).

The following 2° antibodies were used at 1:200 dilution: AlexaFluor 488 anti-chicken IgG, anti-rat IgG, anti-mouse IgG, anti-rabbit IgG, anti-guinea pig IgG; AlexaFluor 543 anti-rat IgG, anti-rabbit IgG, anti-guinea pig IgG, anti-mouse IgG; AlexaFluor 594/546 anti-mouse IgM; AlexaFluor 647 anti-rat IgG, anti-mouse IgG, anti-rabbit IgG, anti-guinea pig IgG and anti-chicken IgY-488 (Invitrogen Life Technologies).

#### Quantification of luminal chitin level in trachea

Average chitin intensity in tracheal segment 7 in *reb* mutant and wild-type trachea were measured using NLS-Element software (Nikon). Ten stage-16 embryos were measured. Fold change between *reb* mutant and wild type was calculated. Average chitin intensity in wild type was used as one fold. Standard errors were shown.

#### Quantification of DT tube length

Stage 16 embryos were stained with chitin probe to label tracheal lumen. Samples were imaged using Nikon confocal microscopy and *z*-stack projections were obtained. Measurements were made using the Nikon NLS Elements program. DT length from tracheal metameres 4-8 (between white arrows in Fig. 2) was measured. Since *Drosophila* embryos may have different embryonic length depending on genotypes, we normalized the DT length to the overall embryonic length. Fold change between *reb* mutant and wild type was calculated. DT length in wild type was used as one fold. Ten embryos were scored per genotype. Standard errors were shown. For statistical analysis of significance, Student *t*-tests were performed to measure differences between different genotypes.
